# The real-world effectiveness of preschoolers wearing masks on campus to prevent respiratory infectious diseases: a cohort study

**DOI:** 10.3389/fpubh.2024.1412884

**Published:** 2024-08-16

**Authors:** Wenhao Yang, Jian Liu, Nanyang Qin

**Affiliations:** ^1^Pediatrics, Shenzhen Samii Medical Center (The Fourth People’s Hospital of Shenzhen), Guangdong, China; ^2^Pediatrics, Shenzhen Baoan People’s Hospital, Guangdong, China; ^3^Intensive care unit (ICU), Shenzhen Samii Medical Center (The Fourth People’s Hospital of Shenzhen), Guangdong, China

**Keywords:** wearing masks, preschoolers, effectiveness, prevent, respiratory infectious diseases

## Abstract

**Background:**

Respiratory infections are common in the pediatric population. Preschoolers, especially those in kindergarten and 3–6 years old, are highly vulnerable to various respiratory infections.

**Objective:**

To investigate the efficacy of indoor mask-wearing in mitigating respiratory infections in preschoolers in a real-world campus setting.

**Methods:**

The study was conducted over a 115-day period in a kindergarten. Eligible children were assigned into study and control groups. The study group wore masks indoors but not outdoors, and the control group did not wear masks in either setting. We used a questionnaire to collect participant information, including age, height, weight, monthly dietary living expenses, family annual income, parent education level, primary caregiver, number of family members, and number of children under 6 years of age in the household. Incidences of clinical respiratory infections were recorded. We calculated the relative risk and analyzed the relationship between mask-wearing and respiratory infections by inter-group comparison, logistic regression, and Cox regression analyses.

**Results:**

A total of 135 preschoolers were included, with 35 and 100 preschoolers in the study and control groups, respectively. Baseline comparisons showed a significant difference in the number of children under 6 years old in the household between the two groups. Mask-wearing did not significantly reduce the risk of respiratory infections (RR = 1.086, 95% CI: 0.713, 1.435). Logistic and Cox regression analyses also showed no significant relationship between mask-wearing and occurrence of respiratory infections after controlling for potential confounders (OR = 0.816, 95% CI: 0.364, 1.826, and HR = 0.845, 95% CI: 0.495, 1.444).

**Conclusion:**

Indoor mask-wearing did not reduce the incidence of respiratory infections in preschoolers in a real-world campus setting. However, this study included a small number of preschoolers and observed them for a short period of time. Preschoolers were instructed to wear masks only when indoors. These factors could lead to bias and limit the generalizability of the study results.

## Introduction

In early 2020, or possibly in the months before, coronavirus disease (COVID-19) broke out and quickly became the most severe global public health concern. Airborne transmissions are reported to be the main route (1, 2), and other routes, such as close contact, should also be emphasized (3, 4). Governments implemented strict non-pharmaceutical interventions (NPIs). Studies reported that the incidence of influenza dropped significantly, and the number of outpatient visits and hospitalizations for respiratory tract infections in adults and children dropped sharply because of COVID-19 prevention and control measures (5, 6). Mask wearing is a key NPI measure. Hypothetically, mask-wearing reduces the exposure to infectious droplets through mechanisms including gravitational settling, strain, interception, diffusion, inertial impact, and electrostatic attraction (7, 8). However, there is no consensus on the effectiveness of mask-wearing to prevent respiratory infections in the real-world setting (9–11).

Respiratory infectious diseases in preschoolers with ages between three and six are an important concern in pediatric outpatient clinics. Kindergartens are often where preschoolers become infected, and this population is highly vulnerable to outbreaks and epidemics of specific diseases, including herpes pharyngitis, hand-foot-mouth disease, influenza, mumps, autumnal diarrhea, and acute gastroenteritis, putting pressure on the healthcare system. Kindergartens should be a key area of concern for reducing infectious diseases in preschoolers.

Researchers have expressed particular interest in understanding the safety and effectiveness of mask-wearing among preschoolers. Social benefits can arise if wearing masks has a positive effect on preventing the spread of respiratory infections within the campus. However, data on whether wearing masks decreases the incidence of respiratory diseases in children is limited. This study is a cohort investigation to assess whether the implementation of wearing masks can decrease the incidence of respiratory infections on campus in real-world setting.

## Methods

### Design

A random selection was made among a publicly funded kindergarten institution in Pingshan District, Shenzhen. They comprised three grades: lower, intermediate, and higher classes. An intermediate student cohort ages four and a half years old on average (median age in the 3- to 6-year-old range) was the most suitable for our study. All preschoolers in intermediate classes received a medical checkup prior to enrollment and were vaccinated in accordance with the Chinese Children’s Immunization Program. The possibility of contracting severe respiratory diseases or congenital diseases is largely eliminated through this program.

In addition to intense exposure during the school day, family factors are also important in assessing additional exposure risk. Children’s susceptibility to infectious diseases is influenced by the following factors: family size, primary caregiver’s education, total family income, number of children in the family, parents’ education level, and students’ food and living expenses. We used questionnaires to eliminate confounding factors outside the school setting. Students were monitored for possible side effects such as allergies and respiratory distress while wearing the mask.

### Ethical considerations

This study was approved by the clinical research ethics committee of Shenzhen Samii Medical Center (The Fourth People’s Hospital of Shenzhen) (KY-2022-04). This study also obtained informed consents from the school administrators, school nurses, teachers, and students’ parents. It was requested that the research data be kept confidential due to the private nature of the information.

### Sample size

A randomized observation of respiratory disease occurrences in an intermediate class at a kindergarten was conducted. This observation took place in the absence of mask usage, showing a disease incidence rate of 0.5. In this study, a control group incidence rate of 0.5 was assumed because data on the protective effect of wearing masks in children under 6 years of age are currently lacking. With a guaranteed power of >0.9, the study group required a minimum sample size of 30, and the control group required a minimum sample size of 90, as calculated using PASS 15.0.

### Clusters

Students were assigned to their classes randomly upon enrollment. To form the study group, one of the four intermediate classes was selected at random, and the remaining three classes formed the control group. The study group consisted of 35 students, and the control group consisted of 100 students, for a total sample size of 135 students at a 1:3 ratio. The sample size ensured that the power was>0.9 and the study’s feasibility. The 1:3 ratio was chosen for several reasons. Having a larger control group can help improve statistical power, allowing more accurate detection of differences between the experimental and control groups. Specifically, a larger control group can provide a more precise baseline estimate and reduce random error and bias (12, 13). Moreover, in studies involving preschool students, a 1:3 ratio of experimental to control groups can help reduce the cost and resources of the study. A smaller study group can reduce the complexity and cost of implementing intervention measures, while a larger control group can ensure the reliability of the data. Similarly, smaller study groups present fewer ethical challenges and are more likely to be supported by schools and parents. Finally, a larger control group can help reduce bias and improve the internal and external validity of the study results, and this design method is widely used in epidemiological studies, especially in observational studies and intervention trials (14).

### Practice

The researcher provided adequate information, regarding the research, to the teachers and parents of the preschoolers before initiating the study to facilitate their comprehension of the research. Training was provided for the school administrators, school nurses, teachers, and all preschoolers in this study. Training included lectures and multimedia learning materials on mask-wearing, as well as skill demonstrations. The training was designed to ensure the nurses and teachers could instruct the preschoolers on how to correctly wear a face mask.

According to the class schedule, parents dropped their children off at the kindergarten where they underwent a health screening administered by the school nurse, which included monitoring their temperature and checking for symptoms of respiratory infections. The students began their classes at 8:00 a.m. and had lunch and a lunch break before being dismissed at 4:30 p.m. The students also had approximately 1 h of outdoor activities in both the morning and afternoon. The class schedule was as follows:

**Table:** 

07:50–08:10	Access to campus (wearing masks)
08:10–09:00	Indoor classes (wearing masks)
09:00–11:40	Outdoor classes (without wearing masks)
11:40–14:40	Lunch and lunch breaks (without wearing masks) Note 1. All students washed their hands before eating under the supervision of teachers. Note 2. Students used convenient beds alternating head and feet for lunch breaks.
14:50–15:30	Outdoor classes (without wearing masks)
15:30–16:10	Indoor classes (wearing masks)
16:10–16:30	Preparing to leave campus (wearing masks)
16:30	Students left campus

The study and the control groups had the same school management system, diet, and environment. The study group began wearing children’s masks (Chinese mask manufacturing standard: YY 0469–2011 “Medical Surgical Masks”) once they entered the school, and they wore children’s masks in indoor classes, but they did not need to wear masks in outdoor classes, lunches, and lunch breaks. The control groups, according to the current policy, did not need to wear masks after entering the school. Normally, a new mask was worn in the morning and in the afternoon. Teachers replaced the masks with new ones if they were wet or contaminated with saliva during the school day.

According to the school administration, teachers disinfected all desks and benches with chlorine disinfectant every day after students left school. Toys were disinfected with chlorine disinfectant once a week. The disinfection was performed for both the study and control groups.

Every morning, school nurses performed physical examination on students as they entered the school. The nurse registered any respiratory infection symptoms, such as coughing, nasal congestion, runny nose, sore throat, or sneezing. The researcher tracked and registered absences resulting from illness or hospital visits. The criteria for the assessment of respiratory diseases are shown in Supplementary Table S1 (9).

Teachers monitored preschoolers for any potential side effects from mask-wearing, such as skin irritation, dyspnea, and headache. If any of these issues occurred, they were reported to the school nurses for evaluation and were recorded. When necessary, the preschoolers were sent to the nearby hospital for further examinations.

## Results

This study was conducted at the end of 2021 for 115 days. The median age of the study participants was 4.4 (IQR: 4.1–4.6). No mask-induced side effects were reported throughout the entire study period.

The study group (*N* = 35) had 46 CRIs, while the control group (*N* = 100) had 121 CRIs. The number of CRIs was greater than the sample size because some of the students had recurring CRI during the period of observation, but there was no significant difference in repeat illness incidence between the two groups (*p* > 0.5) (Table 1). These data are presented as incidence densities, and the incidence density was similar between the two groups with a relative risk (RR) of 1.086 (Table 2).

**Table 1:** Analysis of infection occurrences in the study and control groups.

Number of times infected	Control group (*N* = 100)	Study group (*N* = 35)	χ^2^	*P*
0	45 (45.00%)	16 (45.70%)	7.44	0.282
1	30 (30.00%)	10 (28.60%)		
2	16 (16.00%)	3 (8.60%)		
3	5 (5.00%)	1 (2.90%)		
4	2 (2.00%)	4 (11.40%)		
5	1 (1.00%)	1 (2.90%)		
6	1 (1.00%)	0 (0.00%)		

**Table 2:** Relative risk (RR) in the study and control groups.

	N	Number of CRIs	Incidence densities (persons/day)	RR	95% CI of RR	
					Lower bound	Upper bound
Control group	100	121	0.0105	1.086	0.713	1.435
Study group	35	46	0.0114			

### Comparing basic information across groups

A comparison of demographic characteristics and family structure between the study and control groups (Table 3) did not reveal any significant differences (*p* > 0.5) in age, height, weight, annual family income, primary caregiver’s education, parent’s education, or number of family members. All students were divided into two groups with or without morbidity for comparison to check whether these two factors were confounders. The results (Table 4) showed that there was no statistically significant difference in the monthly cost of food and living (*p* > 0.05), but there was a statistically significant difference in the number of children under 6 years of age (*p* < 0.05). This suggests that the number of children under 6 years of age may have been a confounding factor influencing the results of this study.

**Table 3:** Comparative analysis of basic information of students in the study and control groups.^a^

Variable	Control group (*N* = 100)	Study group (*N* = 35)	Statistic quantity	*P*-value
Age (years)	4.4 (4.1,4.6)	4.3 (4.1,4.8)	W = 1680.5	0.72 ^b^
Weight (kg)	17.80 (16.63,19.28)	18.00 (16.80,19.60)	W = 1714.5	0.86 ^b^
Height (cm)	108 (106,110.5)	108.27 (105,111)	W = 1731.5	0.93 ^b^
Weight/age			χ^2^ = 1.68	0.64 ^c^
Upper	3 (3.00%)	0 (0.00%)		
Upper middle	62 (62.00%)	21 (60.00%)		
Middle lower	34 (34.00%)	14 (40.00%)		
Lower	1 (1.00%)	0 (0.00%)		
Height/age			χ^2^ = 0.31	0.86 ^c^
Upper	3 (3.00%)	1 (2.90%)		
Upper middle	65 (65.00%)	21 (60.00%)		
Middle lower	32 (32.00%)	13 (37.10%)		
Weight/height			χ^2^ = 1.85	0.61 ^c^
Upper	3 (3.00%)	1 (2.90%)		
Upper middle	54 (54.00%)	15 (42.90%)		
Middle lower	42 (42.00%)	19 (54.30%)		
Lower	1 (1.00%)	0 (0.00%)		
Monthly living and eating expenses for students (RMB)			χ^2^ = 10.59	0.03 ^c^
<2,000	39 (39.00%)	12 (34.30%)		
2,000–4,000	42 (42.00%)	20 (57.10%)		
4,000–6,000	17 (17.00%)	0 (0.00%)		
6,000–8,000	1 (1.00%)	2 (5.70%)		
>8,000	1 (1.00%)	1 (2.90%)		
Family’s annual income (RMB)			χ^2^ = 6.37	0.17 ^c^
<100,000	14 (14.00%)	10 (28.60%)		
100,000–300,000	24 (24.00%)	11 (31.40%)		
300,000–500,000	36 (36.00%)	7 (20.00%)		
500,000–1,000,000	24 (24.00%)	6 (17.10%)		
>1,000,000	2 (2.00%)	1 (2.90%)		
Father’s education level			χ^2^ = 1.93	0.59 ^c^
High school	11 (11.00%)	2 (5.70%)		
Junior college degree	11 (11.00%)	5 (14.30%)		
Bachelor’s degree	57 (57.00%)	23 (65.70%)		
Master’s degree	21 (21.00%)	5 (14.30%)		
Mother’s education level			χ^2^ = 1.31	0.86 ^c^
High school	11 (11.00%)	3 (8.60%)		
Junior college degree	23 (23.00%)	10 (28.60%)		
Bachelor’s degree	57 (57.00%)	18 (51.40%)		
Master’s degree	8 (8.00%)	4 (11.40%)		
Doctoral and post-doctoral degree	1 (1.00%)	0 (0.00%)		
Primary caregivers			χ^2^ = 0.87	0.65 ^c^
Babysitter	2 (2.00%)	0 (0.00%)		
Parents	76 (76.00%)	26 (74.30%)		
Grandparents	22 (22.00%)	9 (25.70%)		
Number of family members			χ^2^ = 2.44	0.49 ^c^
<3	17 (17.00%)	5 (14.30%)		
4	40 (40.00%)	16 (45.70%)		
5	28 (28.00%)	12 (34.30%)		
>6	15 (15.00%)	2 (5.70%)		
Number of children under 6 years of age in the household			χ^2^ test	0.02 ^c^
1	70 (70.00%)	17 (48.60%)		
≥2	30 (30.00%)	18 (51.40%)		

a Continuous variables are reported as median and interquartile range, which were analyzed using the rank-sum test because none of these variables had a normal distribution after the normality test. Categorical variables are reported as absolute number and composition ratio, which were analyzed using the chi-square test with chi-square as statistic.

b Wilcoxon rank-sum test.

c χ^2^-test.

**Table 4:** Summary of basic information for students with and without CRIs^a^.

Variable	Incidence		Statistic quantity	*P-*value
	No (*N* = 61)	Yes (*N* = 74)		
Age (years)	4.40 (4.10, 4.65)	4.40 (4.10, 4.70)	W = 2240.5	0.94^b^
Weight (kg)	17.90 (16.75, 19.25)	17.90 (16.65, 19.60)	W = 2239.5	0.94^b^
Height (cm)	108 (106,110)	108.14 (105,111.13)	W = 2209.5	0.83^b^
Weight/age			χ^2^ = 1.64	0.65^c^
Upper	1 (1.60%)	2 (2.70%)		
Upper middle	36 (59.00%)	47 (63.50%)		
Middle lower	23 (37.70%)	25 (33.80%)		
Lower	1 (1.60%)	0 (0.00%)		
Height/age			χ^2^ = 1.48	0.48^c^
Upper	3 (4.90%)	1 (1.40%)		
Upper middle	38 (62.30%)	48 (64.90%)		
Middle lower	20 (32.80%)	25 (33.80%)		
Weight/height			χ^2^ = 1.89	0.60^c^
Upper	2 (3.30%)	2 (2.70%)		
Upper middle	33 (54.10%)	36 (48.60%)		
Middle lower	25 (41.00%)	36 (48.60%)		
Lower	1 (1.60%)	0 (0.00%)		
Monthly living and eating expenses for students (RMB)			χ^2^ = 2.66	0.62^c^
<2,000	22 (36.10%)	29 (39.20%)		
2,000–4,000	30 (49.20%)	32 (43.20%)		
4,000–6,000	7 (11.50%)	10 (13.50%)		
6,000–8,000	2 (3.30%)	1 (1.40%)		
>8,000	0 (0.00%)	2 (2.70%)		
Family’s annual income (RMB)			χ^2^ = 2.08	0.72^c^
<100,000	12 (19.70%)	12 (16.20%)		
100,000–300,000	17 (27.90%)	18 (24.30%)		
300,000–500,000	16 (26.20%)	27 (36.50%)		
500,000–1,000,000	14 (23.00%)	16 (21.60%)		
>1,000,000	2 (3.30%)	1 (1.40%)		
Father’s education level			χ^2^ = 5.23	0.16^c^
High school	3 (4.90%)	10 (13.50%)		
Junior college degree	9 (14.80%)	7 (9.50%)		
Bachelor’s degree	34 (55.70%)	46 (62.20%)		
Master’s degree	15 (24.60%)	11 (14.90%)		
Mother’s education level			χ^2^ = 4.73	0.32^c^
High school	3 (4.90%)	11 (14.90%)		
Junior college degree	17 (27.90%)	16 (21.60%)		
Bachelor’s degree	35 (57.40%)	40 (54.10%)		
Master’s degree	6 (9.80%)	6 (8.10%)		
Doctoral and post-doctoral degree	0 (0.00%)	1 (1.40%)		
Primary caregivers			χ^2^ = 0.02	0.99^c^
Babysitter	1 (1.60%)	1 (1.40%)		
Parents	46 (75.40%)	56 (75.70%)		
Grandparents	14 (23.00%)	17 (23.00%)		
Number of family members			χ^2^ = 1.94	0.59^c^
<3	9 (14.80%)	13 (17.60%)		
4	29 (47.50%)	27 (36.50%)		
5	17 (27.90%)	23 (31.10%)		
>6	6 (9.80%)	11 (14.90%)		
Number of children under 6 years of age in the household			χ^2^ = 4.22	0.03^c^
1	45 (73.80%)	42 (56.80%)		
≥2	16 (26.20%)	32 (43.20%)		

a For continuous variables, which were analyzed using the rank-sum test with the W value as statistic, the normality test showed that none of them followed a normal distribution, so the median (interquartile range) is reported. For categorical variables, both absolute number and composition ratio (in brackets) are reported. The chi-square test was used for statistical analysis with the chi-square value as statistic.

b Wilcoxon rank-sum test.

c χ^2^-test.

### Single-factor and multifactor logistic regression analysis

To investigate the difference in the risk of respiratory infections between the study and control groups without controlling for confounders, univariate logistic regression was conducted. The occurrence of infection was used as the dependent variable, and the results (Table 5) indicated that wearing masks on campus did not reduce the risk of respiratory diseases among students. Furthermore, the variable of the number of children under the age of 6 years was included, and to explore the difference in the risk of infection between the study group and the control group after controlling for the confounding factors, the multivariate logistic regression was established. The results (Table 6) showed that the risk of wearing masks did not change after controlling for the factor of the number of children under the age of 6 years in the household.

**Table 5:** Univariate logistic regression.

Variable	Coefficient of regression	Standard error	Wals value	*P-*value	OR	95% CI	
						Lower bound	Upper bound
Study group (vs. control group)	−0.029	0.394	0.005	0.942	0.972	0.449	2.105
Constant quantity	0.201	0.201	0.997	0.318	1.222		

**Table 6:** Multivariate logistic regression.

Variable	Coefficient of regression	Standard error	Wals value	*P-*value	OR	95% CI of OR	
						Lower bound	Upper bound
Study group (vs. control group)	−0.204	0.411	0.246	0.62	0.816	0.364	1.826
Number of children under 6 years of age in the household = 1 (vs. ≥2)	−0.801	0.383	4.37	0.037	0.449	0.212	0.951
Constant quantity	0.771	0.346	4.967	0.026	2.162		

### Univariate and multivariate COX regression analyses

**Figure 1:**
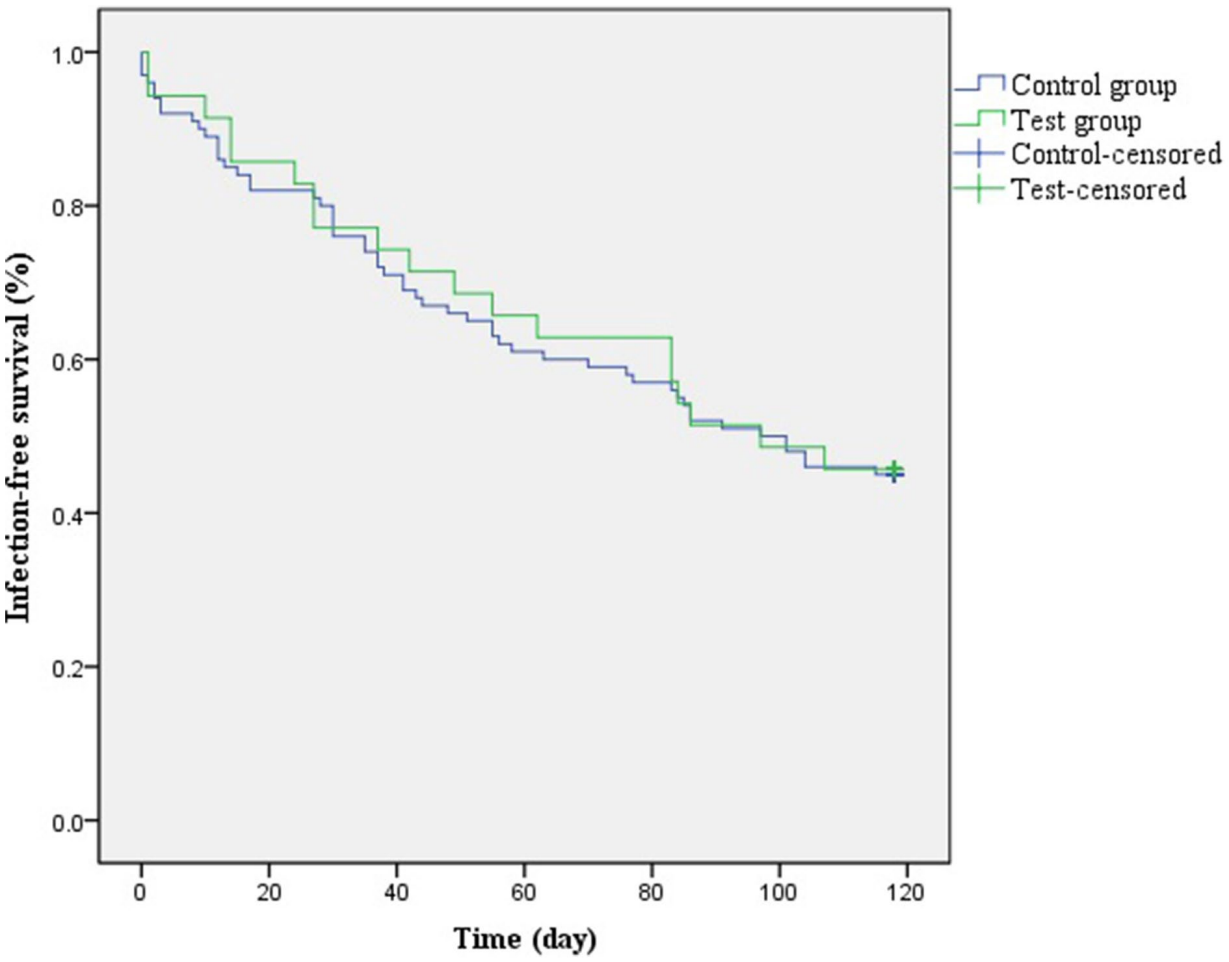
Survival curves in the control and study groups.

A COX regression analysis was performed considering the time from the beginning of the study to the time of the first occurrence of the outcome event for an individual. To explore the differences in the time to the occurrence of the outcome event between the study group and the control groups, the occurrence of infection was used as the outcome variable. The results (Table 7; Figure 1) showed no significant effect of wearing masks on campus on the occurrence of respiratory infections in the students regarding the outcome. Further, the number of children under 6 years of age in the household was included as a baseline variable. A multifactorial COX regression was set up to explore the differences in the time to disease infection between the study and control groups after controlling for confounders. The results (Table 8) showed no change in the outcome after controlling for the number of children under 6 years of age in the household as a risk factor.

**Table 7:** Univariate analysis of time to infection.

Test statistic	Chi-square value	Degree of freedom	*P*-value
Rank-sum ratio test	0.023	1	0.88

**Table 8:** COX multivariate regression analysis.

Variable	Coefficient of regression	Standard error	Wals value	*P-*value	HR	95% CI of HR	
						Lower bound	Upper bound
Study group (vs. control group)	−0.168	0.273	0.379	0.538	0.845	0.495	1.444
Number of children under 6 years of age in the household = 1 (vs. ≥2)	−0.521	0.241	4.673	0.031	0.594	0.37	0.952

## Discussion

In pediatric outpatient and emergency departments in China, young children make up the majority of patients. Most cases consist of respiratory tract infections caused by viruses, including influenza, respiratory syncytial, and parainfluenza (15, 16). Viral transmission occurs primarily through droplet inhalation. The virus can be transmitted from an infected person’s respiratory secretions to a susceptible person through inhalation or contact with droplet-contaminated objects, such as hands, toys, utensils, and clothing. Wearing a mask may reduce the incidence of respiratory infectious diseases (17–20) and is recommended by the World Health Organization (WHO) and U.S. Centers for Disease Control and Prevention (CDC). However, scientific evidence to support the benefits of mask-wearing to prevent respiratory infections remains lacking in the real-world settings.

There was no consensus on the effectiveness of mask-waring to prevent respiratory infections. For example, MacIntyre et al. conducted a trial in 2016, and the results suggested that wearing masks was potentially effective in preventing the spread of influenza; however, the results were not significant and further studies with larger sample sizes are required (9). In 2024, Sandlund et al. performed a systematic review on 22 studies to assess the effectiveness of mask-wearing in children. The results showed that wearing masks did not significantly prevent the occurrence of respiratory infections. This review also noted various bias in the included studies (10). In addition, other studies suggested that adherence to the mask-wearing guideline was the key to its effectiveness (21, 22).

Prior to the start of the study, parents and teachers expressed their skepticism about the feasibility of children wearing masks, aligning with the prevailing public distrust of children using masks correctly. Teachers were concerned that ensuring students wore masks would increase their workload and had concerns regarding safety. However, school managers, teachers, and parents were willing to cooperate with the study after the researchers conducted science education about masks. To obtain rigorous data, we designed this study to ensure high adherence through sufficient training for teachers and school nurses. The study was also conducted under close supervision by the researcher to ensure correct mask-wearing.

The study group wore masks only in the indoor classroom. According to the WHO, wearing masks outdoors is not recommended due to the risk of reduced breathing capacity. Although some studies have demonstrated that mask-wearing for a short period of time did not cause significant physiological damage, data on the prolonged mask wearing in children are lacking (23, 24). Nevertheless, one study reported 59 negative events of wearing masks in school (25). Another study reported discomfort during mask-wearing, overheating, and negative subjective perception of breathing, in 24 children during exertion (26). Several researchers reported concerns about the increased risk of heat stroke with mask-wearing (27). Therefore, in the current study, we focused on the indoor mask-wearing, but did not allow outdoor mask-wearing.

There was also no consensus on the frequency of mask changes. According to the guidelines from the Chinese Center for Disease Control and Prevention, face masks should not be worn for more than 8 h and should be changed every 4 h for occupationally exposed personnel (28). The results of a randomized controlled trial (RCT) on masks to control the transmission of influenza instructed the intervention group to change their masks every 3 h, but no conclusions about the effectiveness of the masks could be drawn because the experiment was terminated early (29). In another RCT, no statistically significant difference was observed between the incidence of influenza in the mask-only group (mask change every 6 h) and no-mask group among students in a school setting. The type of mask used, however, was not specified in that study (30). In the current study, we changed face masks every 4 h (approximately 8 h in school for these children. One new face mask was used in the morning and a new face mask was used in the afternoon). The impact of optimal frequency of mask changes requires further studies.

This study was not limited to a particular pathogen, and it mainly explored the real-world effectiveness of wearing masks on campus. The average age of the preschoolers who participated in this study was 4.4 years old, which was representative of the age group of preschoolers (3–6 years). This included assessing sociological factors to avoid confounding factors and to make the study as objective as possible in terms of the efficacy of wearing masks to prevent respiratory infections in preschoolers. During the follow-up period, some preschoolers had recurrent illnesses. Each illness event was recorded as one occurrence. The incidence density of CRIs in the study and control groups was almost the same, with a RR of 1.086. This indicated that mask-wearing was not effective in reducing the incidence of respiratory infections in these preschoolers.

The main intervention of mask-waring in this study was within the campus. However, the transmission of respiratory infections could be influenced by multiple factors, such as population, social factors, and virology. To control for the confounding factors outside the campus, we incorporated additional characteristics, such as social and family factors. The quality of life and health status of preschoolers can be affected by the family finance resources, such as annual family income and monthly cost of living. The level of parental education was considered since their general medical knowledge and perception could affect hygienic habits of preschoolers, which might influence the occurrence of diseases (31). In the present study, the above factors were not statistically different between the two groups. We found that a statistically significant difference in the number of children under 6 years of age between the two groups. This factor was included in the logistic regression and COX survival analyses as a confounding factor, but it did not affect the study outcome.

Therefore, this study found that indoor mask-wearing did not significantly reduce the risk of respiratory infections among preschoolers in the real-world setting. This result was consistent with previous studies that found no statistically significant effect of wearing masks in preventing respiratory infections (5, 9, 17, 30). This study was conducted during the COVID-19 pandemic. The reduced social interactions associated with the physical distancing measures implemented during this time may account for the low incidence rate in the control group. Until a consensus can be reached on the effectiveness of mask-wearing to prevent the respiratory infections, we still recommend the public to follow the guidelines from various authorities to wear the face mask to potentially reduce the transmission of respiratory infections and protect health. This is especially important during an epidemic.

### Limitations

This study included a small number of preschoolers and observed them for a short period of time. A large-scale study in different regions, seasons, and age groups is needed. In addition, we only studied indoor mask wearing of preschoolers, which could limit the generalizability of our results. Finally, it could be possible that other unmeasured confounders, such as family social activities, could influence the results.

## Conclusion

Indoor mask-wearing did not reduce the occurrence of respiratory infections in preschoolers in a real-world campus setting. Larger multicenter and long-term clinical trials are required to further address the effectiveness of face mask on respiratory infection prevention. Before a definitive consensus can be reached, the preschoolers should still be encouraged to follow the guidelines from different authorities and wear the face mask to potentially reduce the transmission of respiratory infections and protect health.

## Data Availability

The raw data supporting the conclusions of this article will be made available by the authors, without undue reservation.
