# Supplementary material for: The real-world effectiveness of preschoolers wearing masks on campus to prevent respiratory infectious diseases: a cohort study
Source: Front Public Health. 2024 Aug 16;12:1412884. doi: 10.3389/fpubh.2024.1412884 (PMC11361920; doi:10.3389/fpubh.2024.1412884)
Supplement: Supplementary file 1 [file Table_1.DOCX]

**Supplementary materials**

| Table S1 Criteria for the assessment of respiratory diseases ^a^ | |
| --- | --- |
| CRIs diagnosed by a school nurse, pediatrician, or community physician | (1) Two or more respiratory symptoms (cough, nasal congestion, runny nose, sore throat, or sneezing) or one respiratory symptom accompanied by fever (temperature > 37.3°C).  (2) Fever (temperature ≥37.3°C) not accompanied by other symptoms but diagnosed as acute upper respiratory tract infection.  (3) Lower respiratory tract infections such as bronchitis and bronchopneumonia. |

^a^ Between episodes of CRI, with symptoms in remission for at least 72 hours. CRI: clinical respiratory infection.
